# Impact of minimal invasive extracorporeal circulation on systemic inflammatory response – a randomized trial

**DOI:** 10.1186/s13019-024-02903-8

**Published:** 2024-07-03

**Authors:** Deborah Richards Halle, Leila Louise Benhassen, Karsten Lund Søberg, Peter Fast Nielsen, Hans-Henrik Kimose, Adrian Bauer, John Michael Hasenkam, Ivy Susanne Modrau

**Affiliations:** 1 Department of Cardiothoracic and Vascular Surgery, Aarhus University Hospital, Palle Juul-Jensens Boulevard 99, Aarhus N, 8200 Denmark; 2 Department of Clinical Medicine, Aarhus University, Aarhus, Denmark; 3 Dept. of Cardiovascular Perfusion, MediClin Heart Centre Coswig, Coswig, Germany

**Keywords:** Minimal invasive extracorporeal circulation, Systemic inflammatory response, Systemic inflammatory response syndrome, Coronary artery bypass grafting

## Abstract

**Background:**

Extracorporeal circulation causes a systemic inflammatory response, that may cause postoperative haemodynamic instability and end-organ dysfunction. This study aimed to investigate the impact of minimal invasive extracorporeal circulation (MiECC) on the systemic inflammatory response compared with conventional extracorporeal circulation (CECC).

**Methods:**

Patients undergoing coronary artery bypass grafting were randomized to MiECC (*n* = 30) and CECC (*n* = 30). Primary endpoint was tumor necrosis factor-α. Secondary endpoints were other biochemical markers of inflammation (IL1β, IL6 and IL8, C-reactive protein, leukocytes), and markers of inadequate tissue perfusion and tissue damage (lactate dehydrogenase, lactate and creatine kinase-MB). In addition, we registered signs of systemic inflammatory response syndrome, haemodynamic instability, atrial fibrillation, respiratory dysfunction, and infection.

**Results:**

Patients treated with MiECC showed significantly lower levels of tumor necrosis factor-α than CECC during and early after extracorporeal circulation (median: MiECC 3.4 pg/mL; CI 2.2–4.5 vs. CECC 4.6 pg/mL; CI 3.4–5.6; *p* = 0.01). Lower levels of creatine kinase-MB and lactate dehydrogenase suggested less tissue damage. However, we detected no other significant differences in any other markers of inflammation, tissue damage or in any of the clinical outcomes.

**Conclusions:**

Lower levels of TNF-α after MiECC compared with CECC may reflect reduced inflammatory response, although other biochemical markers of inflammation were comparable. Our results suggest better end-organ protection with MiECC compared with CECC. Clinical parameters related to systemic inflammatory response were comparable in this study.

**Clinical registration number:**

NCT03216720.

## Background

Conventional extracorporeal circulation (CECC) stimulates a systemic inflammatory response after cardiac surgery triggering complement activation, pro-inflammatory cytokine secretion and leukocyte activation [1, 2]. The inflammatory response is further exacerbated by surgical trauma, ischaemia–reperfusion injury, blood loss and re-transfusion of shed blood. Consumption of coagulation factors and platelet activation occur simultaneously, further amplifying the inflammatory response [3]. This inflammatory response can provoke cellular damage leading to bleeding disorders and anaemia, acute kidney injury, respiratory failure, haemodynamic instability and neurological complications [4]. TNF-α is a cytokine released from the myocardium secondary to ischemia-reperfusion injury, and it contributes directly to myocardial dysfunction and the pathogenesis of systemic inflammatory response syndrome (SIRS) [5, 6]. Minimal invasive extracorporeal circulation (MiECC) systems have been introduced to attenuate the hazardous consequences of CECC. State-of-the-art type III and IV MiECC-systems [7] incorporate a fully closed circuit, a centrifugal pump, reduced priming volume, low-volume cardioplegia, reduced total surface area, biocompatible surfaces, a membrane oxygenator, a heat exchanger, a venous air removing device, and a cell-saving device to process and reinfuse shed blood. Evidence regarding the impact of MiECC on inflammatory response based on few small RCT’s remains contradictory. Attenuation of inflammation as measured by inflammatory markers is supported by some [1, 4, 8] and rejected by others [9, 10]. Furthermore, the correlation between measured inflammatory markers and the clinical presence of SIRS is also inconsistent [11]. A randomized controlled trial from Remadi et al. [7], investigated postoperative complications in 400 patients when using MiECC compared with CECC. They found a significantly lower incidence of atrial fibrillation, severe renal failure and focal neurologic complications in the MiECC-group compared with CECC. They found significantly lower postoperative levels of C-reactive protein which supported reduced inflammatory response in the MiECC-group. Wiesenack et al. [12] found lower incidence of myocardial infarction, stroke, atrial fibrillation and renal insufficiency, but no correlation to markers of systemic inflammatory response in a retrospective cohort study of 970 patients. Due to these limited and partially conflicting results, further studies are warranted to investigate the systemic inflammatory response associated with extracorporeal circulation during cardiac surgery.

We hypothesised that MiECC would reduce the inflammatory response, as assessed by biochemical markers and clinical signs compared with CECC.

The overall aim of this study was to investigate whether usage of MiECC compared with CECC attenuates the inflammatory response early after coronary artery bypass grafting (CABG). We compared biochemical markers, and clinical manifestations of inflammation in the two settings.

## Methods

### Ethical statement

The current study is a sub-study of the randomized single-center ‘Miniaturized Extracorporeal Circulation Study’ (ClinicalTrials.gov ID: NCT03216720). Study data were collected and managed using REDCap database electronic data capture tools hosted at Aarhus University Hospital [13]. Data were processed and archived according to the guidelines of the Danish Data Protection Agency.

### Study design

**Fig. 1:**
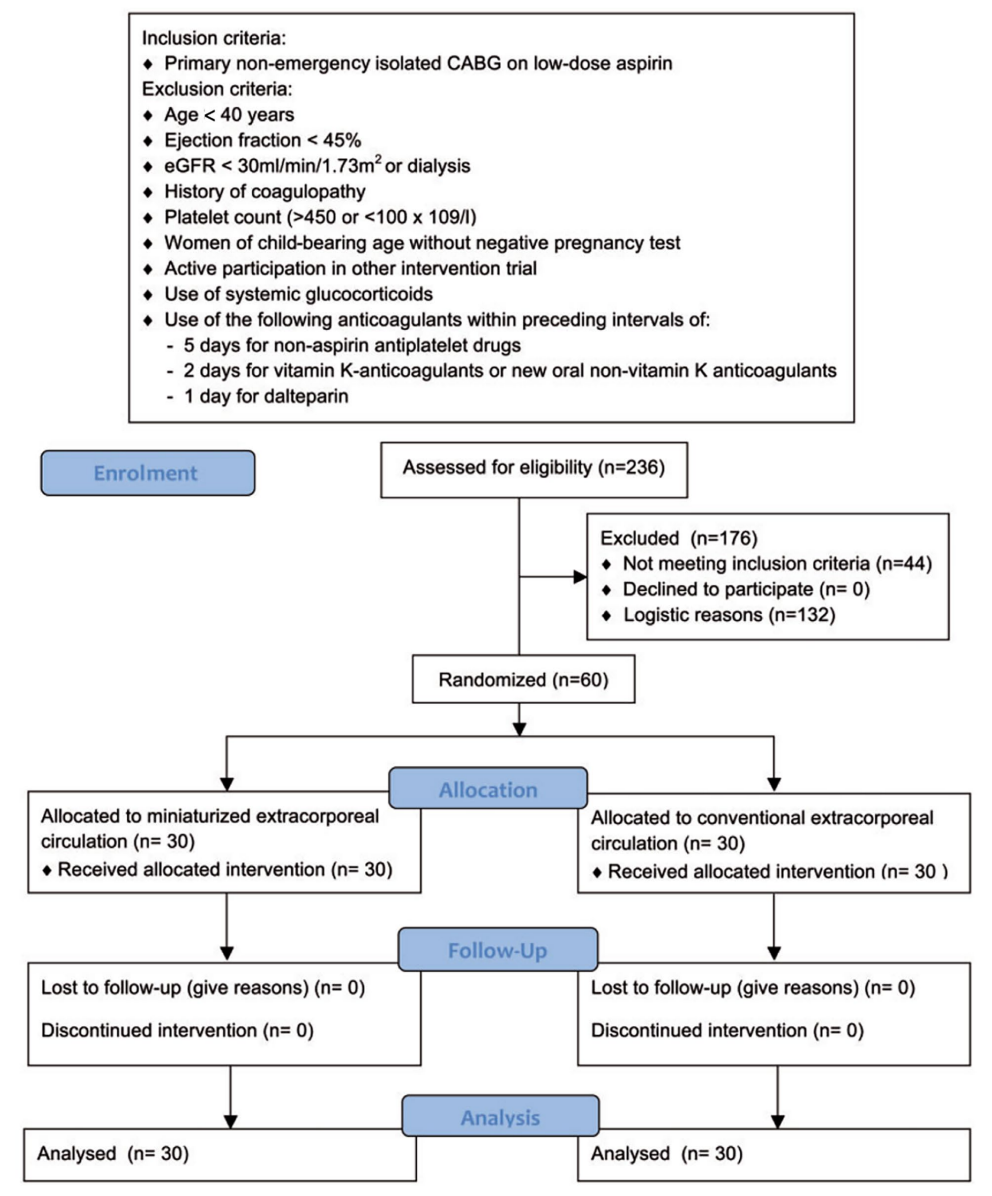
Study CONSORT flow diagram including in- and exclusion criteria. Figure legend: CABG: Coronary Artery Bypass Grafting; eGFR: estimated Glomerular Filtration Rate

Study CONSORT flow diagram including in- and exclusion criteria of this study is depicted in Fig. 1.

60 patients were enrolled from September 28, 2017 to October 31, 2018. All 60 patients undergoing CABG supported by extracorporeal circulation were randomized in a 1:1 ratio to either MiECC or CECC. Patients, medical staff and outcome assessors were blinded, while the operating team inevitably knew the treatment allocation.

### Clinical management

Anaesthetic regimen, operative treatment and postoperative management were comparable as previously described in detail [14, 15]. All procedures were performed by members of the MiECC team at our institution including two surgeons to minimize interpersonal variability. Anaesthesia was induced and maintained with propofol, sufentanil, rocuronium, and sevoflurane. Restrictive fluid management was applied throughout the perioperative period to minimize haemodilution. Intraoperative positioning of the patient and low-dose vasoactive agents were used to maintain a mean arterial pressure between 50 and 80 mmHg during extracorporeal circulation in both groups. Adequacy of the perfusion pump flow was determined by goal directed perfusion parameters (mixed venous oxygen saturation > 65%, and oxygen delivery level above > 270 ml/min/m^2^). We maintained normothermia (> 36 ºC) and haematocrit levels > 25%. Postoperative analgesia regimen included paracetamol, opioids and ketorolac as required. None of the patients received glucocorticoids postoperatively.

### Intervention

#### MiECC

We employed a type III MiECC system as defined by the Minimal Invasive Extracorporeal Circulation International Society [16]. The circuit consisted of polyvinylchloride and silicone tubing coated with a hydrophilic biosurface (Balance^®^ Biosurface, Medtronic International, Tolochenaz, CH), a centrifugal pump (Affinity^™^ CP centrifugal blood pump AP40, Medtronic International, Tolochenaz, CH), an automatic venous air removal device (Affinity^®^ VARD, Medtronic International, Tolochenaz, CH), and an oxygenator with integrated arterial filter and membrane surface area of 2.5 m^2^ (Affinity Fusion^®^, Medtronic International, Tolochenaz, CH). An automated clamp system was mounted downstream of the centrifugal pump to stop circulation in the incidence of air, low flow, or other incidences requiring an emergency pump stop. Ante- and retrograde autologous priming was used to reduce the effective prime volume. Shed blood was collected using a cell-saver (Autolog^®^, Medtronic International, Tolochenaz, CH). The shed blood was processed and washed using sterile Sodium Chloride (NaCl) 0.9% solution and either reinfused to the systemic circulation or available for direct re-transfusion if required. Myocardial protection was accomplished using antegrade intermittent cold (4 °C) blood modified Calafiore cardioplegia every 20 min.

#### CECC

The CECC system utilised an open circuit with a venous reservoir receiving blood from the venous cannula, from the ascending aortic vent, and from the surgical field. The CECC circuit consisted of uncoated polyvinylchloride and silicone tubing and a hard-shell venous reservoir (Costumpack M450311F, Medtronic International, Tolochenaz, CH), a roller pump (Stöckert S5^®^, Munich, Germany), and an oxygenator with integrated arterial filter and a membrane surface of 2.5 m^2^ (Affinity Fusion®, Medtronic International, Tolochenaz, CH). No antegrade or retrograde priming was used. Myocardial protection was accomplished using antegrade intermittent cold (4 °C) Harefield’s blood cardioplegia (Harefield Hospital formulation, IVEX Pharmaceuticals) every 20 min.

### Outcome measures

The primary endpoint of this study was TNF-α. We chose TNF-α as a primary endpoint due to its central role in the inflammatory cascade.

Secondary endpoints were:

- Biomarkers of inflammation (interleukin-1β (IL-1β), interleukin-6 (IL-6), interleukin-8 (IL-8)), C-reactive protein (CRP), white blood cells (WBC))
- Markers of tissue damage and inadequate tissue perfusion (creatine kinase-MB (CK-MB), lactate dehydrogenase (LDH) and blood lactate).
- Clinical manifestations of inflammation including SIRS.

We recorded the following variables reflecting the detrimental side effects of the inflammatory response: (1) postoperative infections as defined by the Centers for Disease Control and Prevention guidelines and Society of Thoracic Surgeons [17, 18]: leg harvest site infection (superficial or deep), superficial sternal wound infection, deep sternal wound infection, and pneumonia. In addition, we registered all other cases requiring antibiotic treatment; (2) new-onset of atrial fibrillation; (3) markers of haemodynamic instability (nadir cardiac index, mean arterial pressure, peak systemic vascular resistance index, requirement of perioperative inotropic support); (4) markers of respiratory dysfunction (duration of mechanical ventilation, hypercapnia, requirement for supplemental oxygen).

In addition, we assessed evidence of SIRS according to American College of Chest Physicians/Society of Critical Care Medicine consensus conference [19] as: (1) temperature greater than 38 °C or less than 36 °C; (2) spontaneous heart rate > 90 beats per min; (3) respiratory rate > 20 per min or pCO2 < 4.3 kPa; and (4) WBC < 4 × 10^9^/l or > 12 × 10^9^/l.

### Laboratory analysis

Venous blood samples were collected at the following time points: “pre-op”, preoperatively; “on heparin” = during ECC, “post-op”= immediately after surgery; “6 hours”= 6–10 hours after surgery, “day 1”, “day 2”, “day 3”, and “day 4” for the following postoperative days. Levels of cytokines (TNF-α, IL1β, IL-6, IL-8) and blood lactate were analysed at all time points up to the first postoperative day in samples drawn from a dedicated line on the central venous catheter. LDH and CK-MB were measured at “6 hours” and “day 1”. We continued measurements of CRP and WBC up to the fourth postoperative day. All blood samples were analysed as previously described [14, 15]. For cytokine analysis (TNF-alpha, IL1-beta, IL-6, IL-8), blood was drawn from an arterial cannula into 3,2% sodium citrate tubes and centrifuged at 3,000 g for 25 minutes at 20°C within 1 hour after blood sampling. Plasma was aliquoted into secondary tubes and frozen at -80°C immediately after centrifugation and stored at -80°C until immediately before analysis. Maximum storage time was 6 months. It was thawed in a water bath at 37°C for 5 minutes. Analysis was performed using MesoScale V-Plex Human Proinflammatory Panel II (Meso Scale Diagnostics, Rockville, Maryland, USA) according to the manufacturer’s instructions.

### Statistical analysis

The sample size estimate was based on a previous study by Fromes et al. [1]. They reported early postoperative TNF-α-levels to be significantly reduced following MiECC compared with CECC (10.1 ± 5.6 ng/l versus 17.8 ± 15.4 ng/l, *p* = 0.002). With a significance level of 0.05, each group required 30 participants to achieve a power of 80%. Data were evaluated for normal distribution by performing the Shapiro-Wilk test. Categorical data are presented as numbers and percentages. Continuous data are presented as mean ± standard deviation when distributed close to normal, otherwise as median (interquartile range). Comparisons between the groups were performed with t-test for continuous outcomes following normal distributions, Fisher’s exact test for all binary outcomes, and otherwise with Wilcoxon–Mann–Whitney test. Comparisons with baseline measurements were performed with paired t-test when values followed normal distribution, and otherwise with Wilcoxon signed rank. P values < 0.05 were considered statistically significant. All statistical analyses were performed using STATA 15^®^ (STATA Corp., College Station, TX, USA).

## Results

Patient demographics, comorbidities, procedural characteristics and perioperative administration of anti-inflammatory drugs were comparable prior to surgery (Table 1). All markers of inflammation were comparable prior to surgery.

**Table 1:** Demographics, comorbidities and procedural characteristics in both groups

Variables	MiECC	CECC	
**Demographics**			
Age (years)	64.6 ± 9.0	68.2 ± 9.2	
Gender (male)	28 (93%)	23 (77%)	
Body mass index (kg/m^2^)	28.2 ± 3.8	27.6 ± 3.9	
Hypercholesterolemia	30 (100%)	28 (93%)	
Arterial hypertension	25 (83%)	25 (83%)	
Diabetes	7 (23%)	9 (30%)	
Insulin-dependent	2 (7%)	3 (10%)	
Non-insulin dependent	5 (17%)	6 (20%)	
Extracardiac arteriopathy^a^	2 (7%)	2 (7%)	
Chronic lung disease^a^	3 (10%)	5 (17%)	
Moderate/severe renal impairment^b^	10 (33%)	16 (53%)	
Ejection fraction < 50%	7 (23%)	8 (27%)	
Acute myocardial infarction < 90 days	5 (17%)	3 (10%)	
History of smoking	20 (67%)	24 (80%)	
History of atrial fibrillation	2 (7%)	1 (3%)	
EuroSCORE II	0.89 (IQR 0.20)	0.99 (IQR 1.02)	
**Indication for revascularization**			
Acute coronary syndrome	10 (33%)	5 (17%)	
Stable angina pectoris	18 (60%)	24 (80%)	
Angina equivalent (dyspnoea)	2 (7%)	1 (3%)	
**Procedural characteristics**			
Total surgery time (min)	186 ± 33	182 ± 39	
ECC time (min)	87 (IQR 25)	76 (IQR 27)	
Aortic cross clamp time (min)	47 (IQR 22)	45 (IQR 26)	
DO_2_ > 270 ml/min/m^2^ during ECC	27 (90%)	24 (80%)	
SvO_2_ > 65% during ECC	27 (90%)	29 (97%)	
Intraoperative Norepinephrine	23 (77%)	24 (80%)	
**Preoperative laboratory findings**			
Haematocrit	44.9 ± 3.7	42.3 ± 5.6	
Haemoglobin (mmol/l)	9.0 ± 0.7	8.8 ± 1.1	
eGFR (ml/min)	87 (IQR 10)	82 (IQR 26)	
CRP (mg/l)	5.8 ± 6.4	5.4 ± 4.5	
Leukocytes (10^9^/l)	7.6 ± 1.9	7.6 ± 1.7	

Continuous data are presented as mean ± standard deviation when distributed close to normal, otherwise as median (interquartile range). CECC = Conventional extracorporeal circulation; CRP = C-reactive protein; DO2 = Oxygen delivery; ECC = Extracorporeal circulation; EuroSCORE II = European system for cardiac operative risk evaluation II; MiECC = Minimal extracorporeal circulation; SvO2 = Mixed venous oxygen saturation; eGFR = Estimated glomerular filtration rate. ^a^Defined according to EuroSCORE II [20]. ^b^Defined as eGFR < 86 ml/min/1.73m2 (CKD-EPI)

### Primary endpoint

**Fig. 2:**
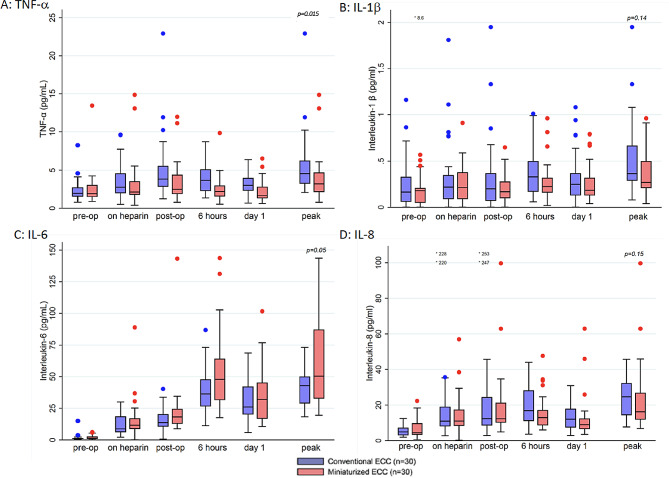
Peri- and postoperative levels of cytokines in both groups. **A**: TNF-α, **B**: IL-1β, **C**: IL-6, **D**: IL-8﻿. Centre line indicates median; box, interquartile range; error bars, upper/lower adjacent values; and dots, outlier values. Time points: “pre-op” = preoperatively; “on heparin” = during ECC, “post-op”= immediately after surgery; “6 h”= 6–10 h after surgery, “day 1”= 1. Postoperative day, “peak” = peak-value of all the values. ECC = ExtraCorporeal Circulation; IL-1β = InterLeukin 1 beta; IL-6 = InterLeukin 6; IL-8 = InterLeukin 8; TNF-α = Tumor Necrosis Factor-α

The course of TNF-α was similar between the two groups with peak values being reached after protamine administration. Peak values of TNF-α were significantly lower in the MiECC group compared with the CECC group (median: MiECC 3.4 pg/mL; CI 2.2–4.5 vs. CECC 4.6 pg/mL; CI 3.4–5.6; *p* = 0.01). In the MiECC group, levels of TNF-α were lower at all time points and dropped to preoperative levels 6 h after surgery while TNF-α remained elevated in the CECC group after 24 h (Fig. 2A).

### Secondary endpoints

#### Biomarkers of inflammation

**Fig. 3:**
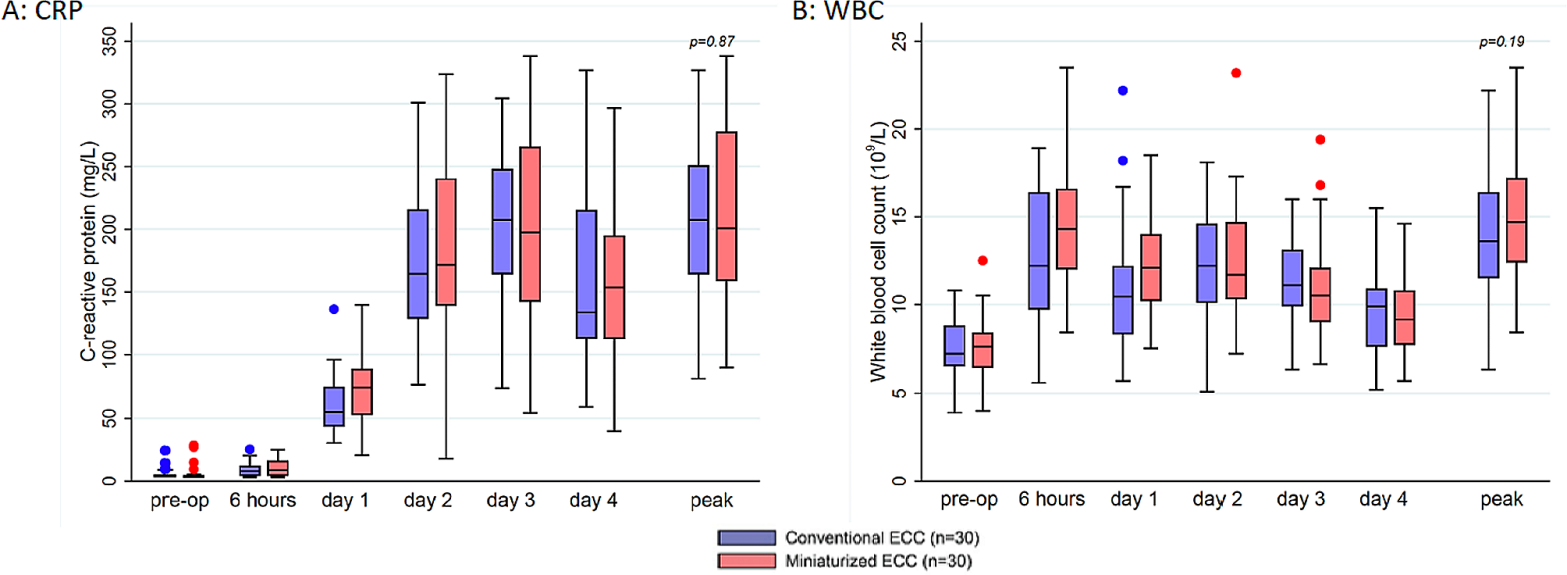
Peri- and postoperative levels of inflammatory markers in both groups. **A**: CRP, **B**: WBC. Centre line indicates median; box, interquartile range; error bars, upper/lower adjacent values; and dots, outlier values. Time points: “pre-op” = preoperative; “6 hours” = 6–10 h after surgery, “day 1” = (1) postoperative day; “day 2” = (2) postoperative day; “day 3” = (3) postoperative day; “day 4” = (4) postoperative day; ECC = ExtraCorporeal Circulation; CRP = C-Reactive Protein; WBC = White Blood Cell

We found comparable perioperative course and peak levels of IL-1β, IL-6, IL-8, WBC and CRP between the groups (Figs. 2 and 3).

#### Markers of tissue damage and inadequate tissue perfusion

Peak-values of CK-MB and LDH up to 24 h postoperatively were significantly lower after MiECC compared with CECC (peak CK-MB (µg/L), MiECC 19.3 (IQR 6.8) vs. CECC 23.5 (IQR 16.5), *p* = 0.03; peak LDH (U/L) MiECC 229 (IQR 80) vs. CECC 380 (IQR 114), <0.001). There were no significant differences in peak blood lactate levels between the two groups during the first 24-hour period (MiECC 1.45 (IQR 0.9) vs. CECC 1.45 (IQR 0.8) mmol/l, *p* = 0.75).

#### Clinical manifestations of inflammation and SIRS

Clinical manifestations of systemic inflammation and haemodynamics were comparable between the groups with exception of significantly higher peak systemic vascular resistance index in the MiECC-group (Table 2). There was no significant difference in any of the clinical outcomes related to inflammation and SIRS. Other clinical outcomes regarding coagulation and renal function have been published previously [14, 15].

**Table 2:** Postoperative markers and clinical signs of inflammation in both groups

Variables	MiECC (*n* = 30)	CECC (*n* = 30)	*p*-value
**SIRS criteria fulfilled**			
At 6 h	14 (47%)	11 (37%)	
Day 1	11(37%)	13 (43%)	
Day 2	16 (53%)	17 (57%)	
Day 3	14 (47%)	16 (53%)	
Day 4	8 (27%)	9 (30%)	
**Infectious complications**, *n* (%)			
Leg harvest site infection	5 (17%)	3 (10%)	0.71
Superficial sternal wound infection	0 (0%)	2 (7%)	0.49
Deep sternal wound infection	0 (0%)	1 (3%)	> 0.99
Pneumonia	5 (17%)	6 (20%)	> 0.99
Other infection requiring antibiotics	4 (13%)	4 (13%)	> 0.99
**New-onset atrial fibrillation (in-hospital)**	12 (40%)	13 (43%)	> 0.99
**Respiratory function**			
Prolonged mechanical ventilation (> 12 h)	0	1 (3%)	> 0.99
Peak partial pressure of carbon dioxide (kPa)	6.4 ± 0.6	6.5 ± 1.3	0.65
**Haemodynamics (up to 24 h postoperative)**			
Nadir Cardiac Index (l/min/m2)	2.0 ± 0.3	1.8 ± 0.3	0.10
Nadir Mean Arterial Pressure (mmHg)	67.8 ± 10.7	69.9 ± 8.50.40	0.40
Peak SVRI (dynes/s/cm^-5^)	1783 ± 374	2063 ± 638	**0.04**
Postoperative requirement Noradrenaline	5 (17%)	6 (20%)	> 0.99

Data presented as number (percentage) or mean ± standard deviation. CECC = Conventional extracorporeal circulation; MiECC = Minimal extracorporeal circulation; SIRS = Systemic inflammatory response syndrome; SVRI = Systemic vascular resistance index

## Discussion

This randomized controlled clinical trial offers a comprehensive investigation of the impact of MiECC on peri- and postoperative systemic inflammatory response compared with CECC. Our study examined a wide range of biochemical and clinical markers of inflammation in patients undergoing CABG. We demonstrated significantly reduced levels of TNF-α early after MiECC as compared with CECC. This result may reflect attenuated inflammatory response but was not supported by other biochemical or clinical findings including the clinical presence of SIRS. An explanation for the observed difference in TNF-α could be due to the use of direct retransfusion with the CECC-system, which contains activated shed mediastinal blood, that triggers blood cell activation and contains high levels of TNF-α [21]. With the MiECC-system, a cell-saver device is used, that washes the blood by eliminating fat, white blood cells and cytokines. It has previously been shown, that by using a cell-saver device with MiECC, the elimination of TNF-α is increased compared with direct retransfusion with CECC [21, 22].

In line with previous studies, systemic TNF-α was elevated when weaning off extracorporeal circulation and in the first hours postoperatively [1, 23, 24].

This study did not demonstrate significant differences in the increase of the other cytokines IL-β, IL-6, and IL-8. Our finding of comparable levels of IL-6 early after MiECC and CECC is consistent with the findings of other studies [9, 25]. In the study by Fromes et al. [1], the pro-inflammatory cytokine IL-6, which also has been reported to be a reliable biomarker of cardiac dysfunction and myocardial damage [26], was significantly lower in the early postoperative stages after MiECC compared with CECC. A possible explanation for the lack of difference between the groups in the current study could be the effect of the volatile anaesthetic agent sevoflurane on the immune response. Sevoflurane has been shown to decrease levels of IL-6 and IL-8 [27]. In our study, the consequent use of sevoflurane may have resulted in low levels of IL-6 and IL-8 without detectable differences between the groups. Fromes et al. [1] did not report which anaesthetic regimen was used in their study. Another explanation could be the different timepoints for measuring the interleukins. In a RCT from Baumbach et al. [25], they investigated the inflammatory response between MiECC and CECC in patients undergoing minimally invasive aortic- or mitral valve procedures. The time course of circulating interleukin-6 levels showed momentarily significantly higher levels in the CECC group one hour after the operation. In accordance with our results, the levels of IL-6 were comparable at all other time-points.

In our study, we demonstrated significantly lower levels of CK-MB and LDH after MiECC compared with CECC. These results are consistent with those of other studies [28, 29]. As in the case with TNF-α, shed mediastinal blood contains high levels of CK-MB and LDH [28, 29]. With direct retransfusion of shed mediastinal blood with CECC, these enzymes reach the circulation and elevate the systemic concentration [28, 29]. MiECC also showed a reduction in haemodilution in the perioperative phase, thus optimising end-organ perfusion during surgery and postoperatively, and reducing the inflammatory response potentially caused by poor oxygen delivery [30]. Using a cell-saver instead of a cardiotomy suction and using biocompatible, are some elements of the MiECC circuit that may contribute to the notable difference in haemolysis and improved end-organ protection as indicated by CK-MB and LDH-levels.

The elevated TNF-α values detected in the CECC-group were without any significant correlations to clinical manifestations or any adverse outcomes. Reducing circulating levels of TNF-α during cardiac surgery may potentially minimise the adverse effects associated with elevated pro-inflammatory cytokines and SIRS. Proinflammatory cytokines such as TNF-α, IL-1β, IL-6 and IL-8, play a pivotal role in stimulating the inflammatory process, with plasma concentrations of specific cytokines, such as IL-1β and IL-6 also predictive of outcome in critically ill patient groups [31]. A study by Squiccimaro et al. [32] showed that 28.3% of patients undergoing cardiac surgery using CECC fulfilled the SIRS criteria in the first 24 h postoperatively. In our study, up to 43% of patients fulfilled the SIRS criteria within the first 24-hour period in both groups, but there was no significant difference between the two groups.

## Conclusions

In conclusion, we found lower levels of TNF-α after minimal invasive extracorporeal circulation (MiECC) compared with conventional extracorporeal circulation (CECC), which may signify reduced inflammatory response, although other biomarkers of systemic inflammatory response were comparable. CK-MB and LDH were also reduced, suggesting better end-organ protection after MiECC compared with CECC. No differences in any clinical parameters related to SIRS were detected in this study.

### Study strengths and limitations

The key strength of this study is its comprehensive evaluation of the systemic inflammatory response after CABG with MiECC compared with CECC in a randomized RCT. Our results can be attributed to the ECC system used, as surgical procedure, anaesthesia, patient selection and postoperative management were comparable between the groups. Postoperative blinding of the treating staff and investigators for outcome assessment and data analysis warranted a low risk of confounding.

However, a number of limitations need to be considered. Per-protocol, this relatively small study was designed to evaluate the difference of TNF-α levels between the groups as a surrogate marker for inflammatory response. The study was not sufficiently powered to assess clinical manifestations of systemic inflammation. Despite its exploratory nature, this study offers valuable insight into the impact of MiECC on the systemic inflammatory response. We chose to compare the full concept of MiECC to CECC as routinely practiced at our department. From this current study, we can therefore not elaborate on the impact of the individual components of the MiECC concept.

We identified no significant differences between the groups in any other biochemical marker of inflammation than TNF-α nor any clinical manifestations. These findings indicate only mild attenuation of the systemic inflammatory response after CABG with MiECC compared with CECC. CABG surgery represents the main area of application for MiECC worldwide. Our results suggest that large confirmatory studies would be required to demonstrate clinical superiority with regards to the adverse systemic effects triggered by systemic inflammatory response in this low-risk patient group and correlating the clinical manifestations of inflammation with biomarkers of systemic inflammatory response. The study is limited by the lack of information on which components of the ECC systems trigger the inflammatory response. Further studies with more focus on the mechanisms that promote inflammation are therefore, suggested.

## Data Availability

No datasets were generated or analysed during the current study.

## Abbreviations

CABG: Coronary artery bypass grafting
CECC: Conventional extracorporeal circulation
CK-MB: Creatine kinase-MB
CRP: C-reactive protein
IL-1β: Interleukin-1β
IL-6: Interleukin-6
IL-8: Interleukin-8
LDH: Lactate dehydrogenase
MiECC: Minimal invasive extracorporeal circulation
TNF-α: Tumor necrosis factorα
SIRS: Systemic inflammatory response syndrome
WBC: White blood cells
